# Resection of inferior vena cava, abdominal aorta, bilateral common iliac arteries, and bilateral partial external iliac arteries with artificial vessel replacement during radical endometrial cancer surgery: a case report

**DOI:** 10.1186/s12905-022-02120-2

**Published:** 2022-12-28

**Authors:** Linlin Yang, Hongying Yang, He Zhao, Zaoxiu Hu, Zhenglei Shen, Lingfeng Zhao, Shufen Tan, Lei Zhu, Ruolan Xu, Hui Liu, Chunyan Ding, Yan Qin, Yanfei Zhao

**Affiliations:** Department of Gynaecology, Yunnan Cancer Hospital (The Third Affiliated Hospital of Kunming Medical University), 650118 Kunming, China

**Keywords:** Endometrial cancer, Radical endometrial cancer surgery, Artificial vessel replacement, Lymph node metastasis

## Abstract

**Background:**

Endometrial carcinoma (EC) is a common malignant tumor of the female reproductive system, often accompanied by lymph node metastasis. Artificial vascular implantation is a common surgical treatment for mediastinal tumors and abdominal aortic aneurysms but is rarely used in gynecological surgery.

**Case presentation:**

A 54-year-old female patient was first admitted to the hospital in January 2018 due to “irregular vaginal bleeding over 3 months”. CT showed a mass in the uterine cavity, and several swollen lymph nodes in the retroperitoneum and pelvic cavity. The initial diagnosis was an endometrial malignant tumor. We performed radical endometrial cancer surgery with parallel resection of inferior vena cava, abdominal aorta, bilateral common iliac arteries, bilateral external iliac arteries, and artificial vessel replacement, which was successful, with good postoperative recovery and no lesion progression at 3 years postoperative follow-up.

**Conclusion:**

This is an early case of gynecological clinical use of prostheses. Through multidisciplinary cooperation, the surgical resection rate of patients with EC in radical surgery was improved without serious fatal complications and achieved a high long-term postoperative survival rate.

## Background

Endometrial cancer (EC) is the most common malignant tumor of the female reproductive system and is generally classified into two types [1]. Type I is the most common, representing more than 70% of cases and is known as endometrioid adenocarcinoma. Type II tumors are of papillary serous or clear cell histologic type. They have carried a poor prognosis and have a high risk of relapse and metastasis [2]. Risk factors for EC include high levels of estrogen (obesity, diabetes, and high-fat diets), early menarche, infertility, delayed menopause, Lynch syndrome, advanced age (55 years or older), and the use of hormone replacement and tamoxifen. Importantly, lymph node metastasis is a common mode of distant metastasis in gynecologic tumors and an independent risk factor for poor patient prognosis [3–5].

Vaginal bleeding is the most common clinical presentation of EC in postmenopausal women [6], in more than 90% of EC cases, women experience intermenstrual or postmenstrual uterine bleeding [7]. The preferred treatment for EC is surgery, including total hysterectomy, bilateral salpingo-oophorectomy, and lymph node dissection [8]. In recent years, the use of artificial vessel replacement surgery in EC clinicals has developed rapidly. We report an early case in which we resected the inferior vena cava, abdominal aorta, bilateral common iliac arteries and bilateral external iliac arteries and veins, and performed artificial vessel replacement during radical surgery for EC in which enlarged lymph nodes invaded the abdominal aorta, inferior vena cava, common iliac arteries and some external iliac arteries and veins.

## Case presentation

**Fig. 1:**
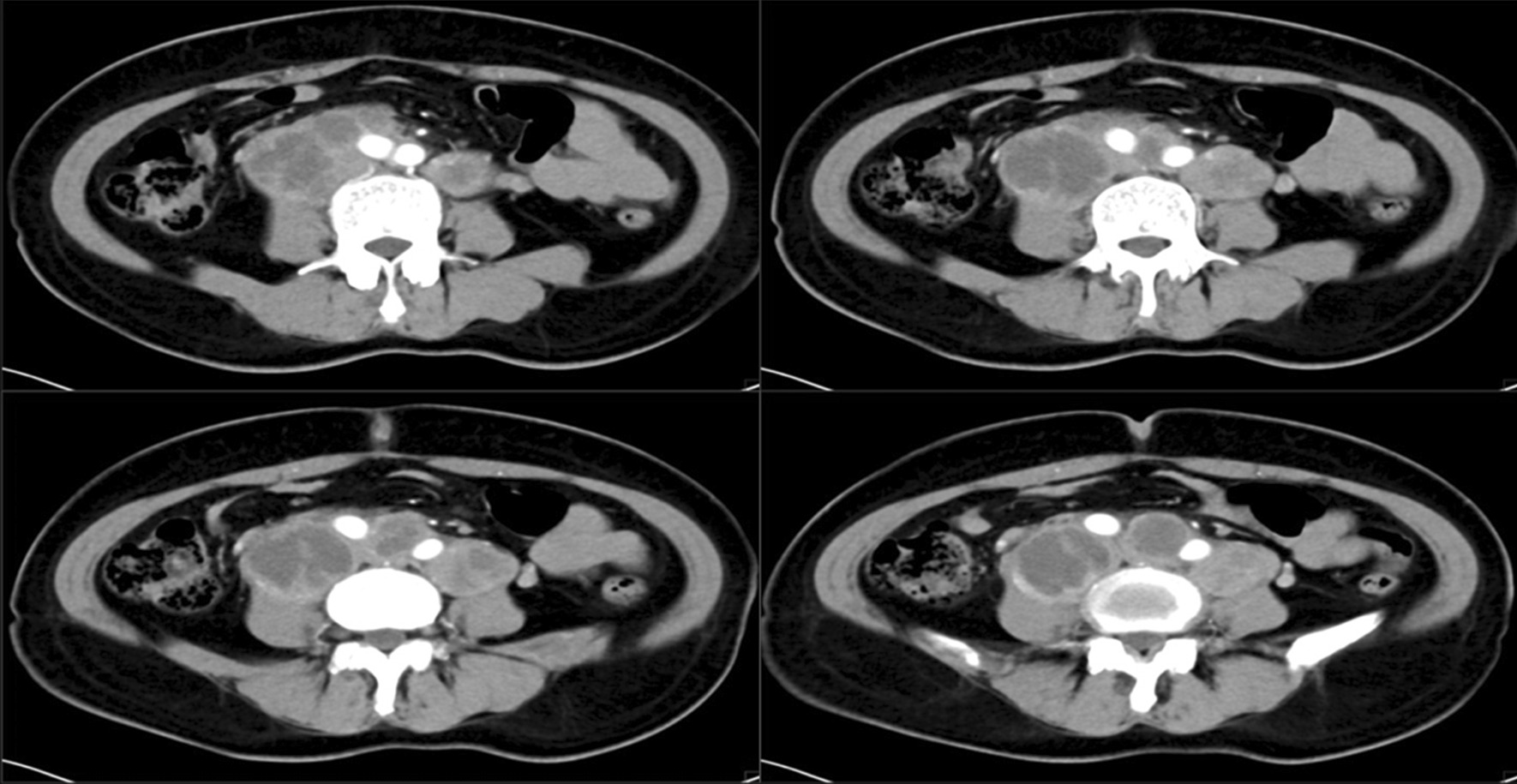
Computed tomography (CT) of the endometrial. Multiple enlarged lymph nodes of varying sizes are seen in the retroperitoneum, adherent to and surrounding the retroperitoneal vessels

**Fig. 2:**
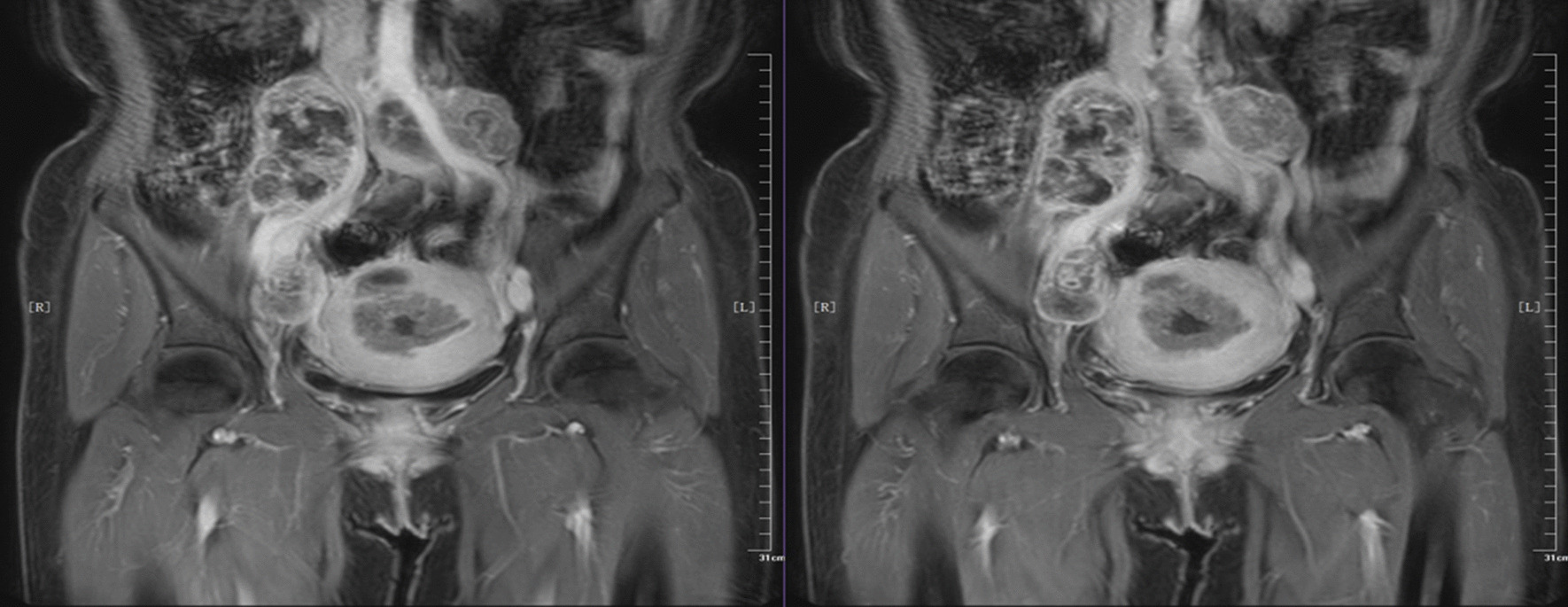
Magnetic resonance imaging (MRI) of the endometrial. Multiple lymph nodes were enlarged retroperitoneally and adjacent to the right iliac vessels, which were adherent and wrapped around the vessels

A 54-year-old female patient, Han nationality, BMI: 24.242, History of hypertension in the past, blood pressure up to 150/100 mmHg. In 2016–2018 years, Zhenju antihypertensive tablets to control blood pressure. Stop the drug half a year before surgery by herself-reported. Preoperative paclitaxel + cisplatin two-stage chemotherapy, Nothing special. The patient is admitted to the hospital first time, on January 15, 2018, with “irregular vaginal bleeding for more than 3 months”. Gynecological examination found that the patient’s cervical canal was thickened with a smooth surface and the neck was translucent with no obvious tumor lesion. The uterine body was enlarged as in the third month of pregnancy, with a smooth surface, and the rest of the examination was unremarkable. Computed tomography (CT) results revealed that there was a mass in the uterine cavity, and the possibility of malignant EC was considered to be high (Fig. 1). MRI confirmed multiple enlarged lymph nodes in the retroperitoneum and pelvis, it may be that extensive lymph node metastasis. The preliminary diagnosis was an endometrial malignant tumor (Fig. 2). Because the patient’s abdominopelvic lymph nodes were large, adhered to blood vessels, and poorly demarcated, it was impossible to cut them cleanly. The neoadjuvant chemotherapy was recommended, after a discussion with MDT experts in the hospital.

The patient underwent 2 courses in postoperative chemotherapy with “paclitaxel liposome + cisplatin” at Yunnan Cancer Hospital, on January 24, 2018, and March 3, 2018. Tumor marker tests on May 8, 2018: glycoantigen-125 84.05 KU/L; glycoantigen-199 140.40 KU/L; human epitope-4 116.90 pmol/L. Human epithelial protein-4 116.90 pmol/L. The Abdominal CT examination result indicated that multiple soft tissue masses of variable size in the mid-lower abdominal retroperitoneum and bilateral parietal iliac vessels, adhering to adjacent vessels, about 4.8 × 4.3 cm in size, mostly considered metastatic lymph nodes, which were enlarged compared to the previous partial lesions. The patient’s tumor markers were higher than in January, no significant changes were seen in the lesion on imaging, and the lymph node was partially enlarged and adhered to blood vessels. There was a high possibility of intraoperative damage to blood vessels if surgery was performed. Because of the difficulty of the operation and the high risk of bleeding, the patient and family fully communicated with each other and agreed to the operation.

**Fig. 3:**
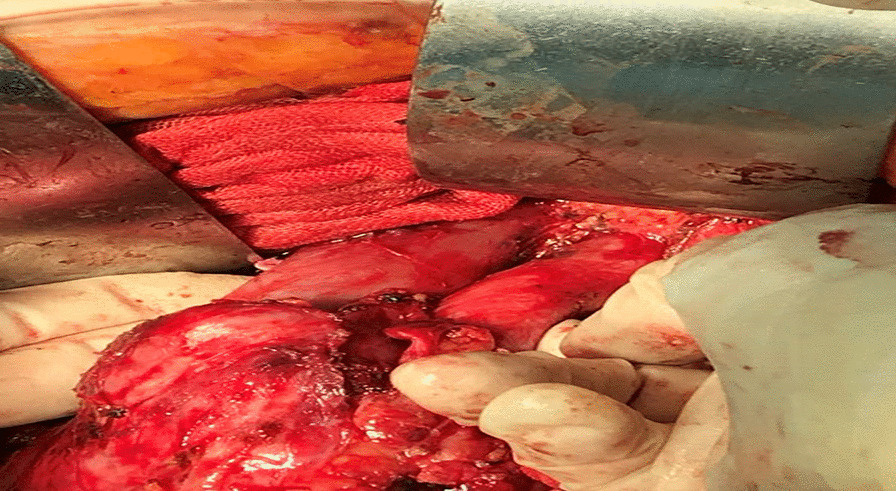
The tumor encloses blood vessels

Intraoperative exploration revealed enlarged lymph nodes in the apex of the abdominal aorta, approximately 13 × 10 × 6 cm. The inferior vena cava and the mural abdominal aorta were surrounded by lymph nodes. The lymph nodes circumferentially surrounded the inferior vena cava and the apex of the abdominal aorta, with the upper pole located below the renal vein and the lower pole located in the anterior sacral region, and both sides surrounded the bilateral common iliac arteries and part of the external iliac artery. The tumor invaded the vessel wall causing the invaded vessels could not be preserved. The right pelvic lymph nodes were enlarged, with the largest located at the right foramen ovale, about 8 × 6 × 4 cm, and encircling the right internal iliac artery. The uterus was enlarged, about 10 × 9 × 7 cm in size, with a smooth surface, normal appearance of the bilateral adnexa, and marked thickening of bilateral main and sacral ligaments. The uterus was dissected and the uterine cavity was seen to be full of lesions, about 7 × 6 × 4 cm, depth of invasion (Fig. 3).

**Fig. 4:**
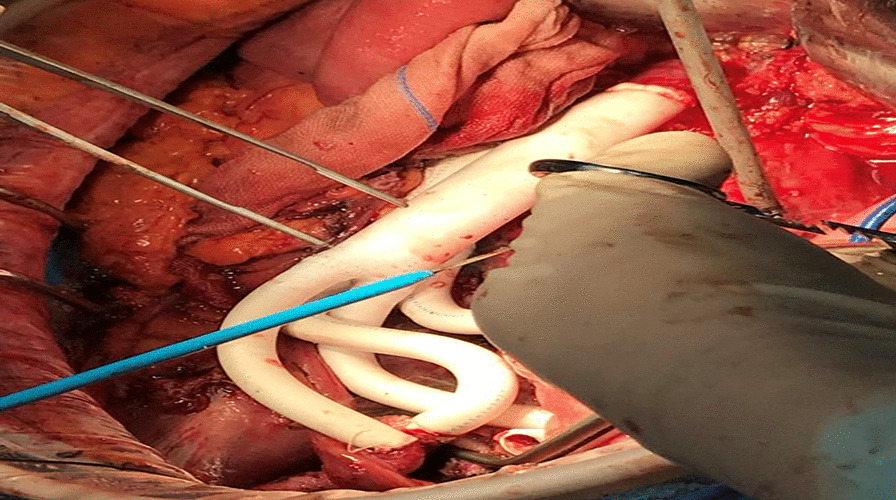
Intraoperative vascular replacement

There was no free of tumor-invasive vessels in the inferior vena cava and the proximal end of the abdominal aorta, and 4 cm from the edge of the tumor, the bilateral external iliac arteries, internal iliac arteries, bilateral external iliac veins, and distal end of internal iliac veins were free, the right internal iliac artery and internal iliac vein were ligated, the left internal iliac vein was ligated, the ends of each free vessel were clamped, the blood flow was blocked, and the inferior vena cava, abdominal aorta, bilateral external iliac arteries and Bilateral external iliac veins (Fig. 4). The operation lasted 10 h intermittently blocking the abdominal aorta for 15 min and 33 s, blood loss 2950 ml, intraoperative transfusion of suspended red blood cells: 9.5 u, frozen plasma: 900 ml, and return to the surgical ICU after the operation.

The patient developed hypoproteinemia, anemia, hypokalemia, hypocalcemia, etc., and the symptomatic treatment improved. After the operation, the patient was in a hypercoagulable state, and dabigatran ester capsule and warfarin were given to prevent thrombosis. The reexamination of blood vessel B ultrasound showed that the proximal end of the left external iliac artery artificial vessel could not be clearly displayed, and the distal end of the external iliac artery was filled with abnormal moderate echo, which did not rule out the possibility of external complete thrombosis. After vascular surgery consultation, it was agreed to inject 4000 IU BID of enoxaparin sodium subcutaneously for anticoagulation treatment. After that, the thrombosis improved and the condition was stable. Injection of cefoperadone sodium tazobactam 2.25 g q8h combined with metronidazole sodium chloride injection 500 mg q12h for anti-infective treatment. On the 5th postoperative day, the patient’s lower limbs weakness. The ultrasound examination discovered that the lumen of the left common iliac artery was indistinct, and the lumen of the external iliac artery was filled with abnormal moderate echogenicity. The nature of which was to be investigated, excluding the possibility of complete thrombosis. The patient was discharged 13 days after surgery.

Based on the postoperative pathology report, the patient was diagnosed as stage IIIC2 medium to highly differentiated endometrioid adenocarcinoma of the uterus. The specific pathological results are as follows: (extensive whole uterus) adenocarcinoma, inclined to endometrioid adenocarcinoma, moderate-highly differentiated with a lot of squamous, cancerous tissue infiltration greater than 1/2 myometrium; cancerous tissue was seen in the endocervical os, cancerous tissue infiltration greater than 1/3 and less than 2/3 layers of cervical fibromuscular wall; (right internal iliac vessel, right external iliac artery stump) microscopically showed vessel wall and fibrous tissue, no cancerous tissue was seen under the microscope. Microscopically, the abdominal aortic lymph nodes are metastatic malignancies with vascular inclusion and carcinoma infiltration.

Overall, After surgery, the patient recovered well. The underwent one course of chemotherapy with “paclitaxel + carboplatin” regimen on July 18, 2018. The TOMO radiotherapy irradiation (DT 45 Gy/25F/35d; DT 180 cGy/F) from July 18 to August 24, 2018. Post-loading treatment of the vaginal stump (12 Gy/2F) was performed on August 15 and August 22, 2018. Four courses of chemotherapy with “paclitaxel + carboplatin” were administered on September 19, 2018, October 23, 2018, November 20, 2018 and December 20, 2018, respectively. The patient was followed up regularly in the external hospital, which revealed no sign of recurrence and metastasis at the cut-off date. The patient no progression in this case for 3 years after surgery indicates an excellent prognosis.

## Discussion

### Surgical problems

EC has the 2nd highest mortality rate of malignant tumors of the female reproductive system in developed countries, and there are risks of bleeding, organ damage, and tumor dissemination when reoperating for recurrence of malignant tumors after surgery [5]. The main treatment for EC is total hysterectomy and bilateral salpingectomy. Furthermore, radiation and chemotherapy can also play a role in the treatment [8]. Aortic lymph node status is a serious prognostic factor for patients with endometrial cancer. Pelvic and paraaortic lymph node dissection is a considerable part of the surgical treatment of gynecological malignant tumors, it aims to reduce the tumor load by defining the lymph node status to guide the choice of treatment plans, or by removing positive lymph nodes to ultimately improve the patient's quality of life [9, 10]. Research analysis indicated that robot lymphadenectomy has less blood loss and shorter hospital stays than laparoscopic lymphadenectomy, and both methods are effective choices for lymphadenectomy [11]. Preoperative analysis and surgical planning are essential before surgical lymphadenectomy. The meta-analysis discovered that 3D-transvaginal ultrasound and magnetic resonance imaging (MRI) have good diagnostic accuracy in detecting preoperative staging and surgical planning of endometrial cancer patients [12]. Positron emission tomography-computed tomography (PET-CT) has also been proven to have excellent preoperative staging and lymph node metastasis diagnostic performance [13]. In the early stage of this study, abdominal CT revealed that there was a mass in the uterine cavity, which was likely to be malignant EC. Subsequently, multiple enlarged lymph nodes in the retroperitoneum and pelvis were found by MRI, which was initially diagnosed as an endometrial malignant tumor. Since the abdominal lymphadenopathy is swollen and attached to the blood vessels of the patient, there is a high possibility of vascular injury and bleeding during the operation. Referring to previous studies, it is considered that laparoscopic lymph node reduction with Yasagil forceps is an effective choice to avoid potentially serious complications during vascular surgery [14], so vascular replacement surgery is considered in this case.

Previously, malignant tumor invasion of vital vessels that could not be resected was considered a contraindication to surgery, but artificial vessel replacement has provided surgical opportunities to improved survival rates for patients with large vessel invasion [15–17]. Among them, Jian successfully performed tumor resection and reconstructed the abdominal aorta and inferior vena cava through externally supported polytetrafluoroethylene vessels [18]. Takashi reported the replacement of an aortic arch aneurysm with 3-branched artificial vessels under independent cerebral and systemic extracorporeal circulation [19]. Kanyu reported a case of simultaneous anastomotic site septum formation surgery and prosthetic vessel replacement to ensure blood outflow access [20]. Xiaoqi proceeds with a complex surgery with complete resection of the mass, IVC with artificial vessel replacement, total hepatectomy, and bilateral nephrectomy with liver and kidney autotransplantation [21]. However, radical surgery with simultaneous resection of the inferior vena cava, abdominal aorta, bilateral common iliac arteries, and bilateral external iliac arteries and veins treated with parallel prosthetic vascular replacement has not been reported.

Artificial vascular implantation is a common treatment procedure in surgery for mediastinal tumors and abdominal aortic aneurysms and is rarely used in gynecologic surgery. The patient was admitted to the hospital in 2018 with a late tumor stage, extensive metastatic lymph node lesions, and invasion of peripheral blood vessels. The prosthesis was not as common as it is now, and this was an early case of clinical use of prostheses. In this case, the vascular surgeon was asked to assist with this multidisciplinary collaboration, which improved the surgical resection rate of EC patients in radical endometrial surgery without serious fatal complications and obtained a high postoperative long-term survival rate, which can be recommended in the treatment of gynecologic surgery.

In this case, the patient was first admitted to the hospital because of the adhesion of the enlarged lymph node next to the abdominal aorta to the surrounding blood vessels, which was difficult to be completely removed surgically, so neoadjuvant chemotherapy was administered. After chemotherapy, there was no significant change in the adhesion between the tumor and the blood vessels. If the mass was forcibly removed, it might cause vascular damage and endanger the patient's life. If the tumor could not be completely removed, the residual lesion would soon recur. After consulting with vascular surgery, an intraoperative vascular replacement could be considered. This case was very successful, and the postoperative complications were properly managed in a timely manner. The patient recovered well and became the first gynecologic malignancy patient in China with a large number of vessels replaced in the initial tumor reduction surgery, a wide range of metastatic lymph node lesions removed, and a tumor-free survival at the follow-up to date.

### Molecular typing

With the molecular staging scheme of EC proposed by The Tumor Genetic Atlas (TCGA) in 2013, molecular staging has been further applied and improved in clinical practice. Molecular typing can not only accurately predict prognosis, but also guide precise treatment, and has high consistency in diagnosis in different hospitals or different regions, which is increasingly recognized by gynecologic oncologists. Endometrial carcinoma is divided into four different molecular subtypes: POLE acculturated; Microsatellite instability hypermutated (MSI-H); Copy number abnormalities-low (CN-L), and Copy number abnormalities-high (CN-H). Literature has reported that MSI exists in many types of solid tumors, such as EC, colorectal cancer, and lung cancer [22], and is also related to Lynch and sporadic endometrial carcinoma [23]. In TCGA studies, MSI high mutants accounted for about 30% of EC, 28.6% of low-grade EEC and 54.3% of high-grade EEC belonged to MSI type. At the molecular level, these tumor characteristic mutations included PTEN (88%), RPL22 (33%), KRAS (35%), PIK3CA (54%), PIK3R1 (40%), and ARID1A (37%) with high TMB levels (18 × 10^6^ Muts/Mb) and certain TIL [24]. Most of these tumors have a good prognosis. In this case, there was no progress in the 3-year follow-up after surgery. The patient was operated on in 2018. At that time, our hospital was unable to perform molecular genotyping and gene testing, and the patient underwent pathology. At present, the patient's MLH1 (–), MSH2 ( +), MSH6 ( +), PMS (–) and the molecular diagnosis was MSI-H, indicating a good prognosis.

## Data Availability

Data sharing is not applicable to this article as no datasets were generated or analyzed during the current study.
